# ChatGPT-4o with faculty guidance outperforms AI-only and traditional learning in ultrasonography training: a randomized trial

**DOI:** 10.3389/fdgth.2026.1772965

**Published:** 2026-03-06

**Authors:** Dao-Rong Hong, Chun-Yan Huang, Jiu Gao

**Affiliations:** 1 Department of Ultrasonography, The Second Affiliated Hospital of Fujian Medical University, Quanzhou, Fujian, China; 2 Department of General Practice, The Second Affiliated Hospital of Fujian Medical University, Quanzhou, Fujian, China

**Keywords:** AI, blended learning, ChatGPT-4o, medical education, ultrasound education

## Abstract

**Background:**

Ultrasonography training for residents is challenging owing to its operator-dependent nature and difficulties in mastering subtle image interpretation. Multimodal large language models like ChatGPT-4o enable efficient knowledge retrieval but show marked limitations in static ultrasonography image analysis.

**Methods:**

In this prospective, single-centre randomized controlled trial, 45 first-year ultrasonography residents were randomly allocated to control (traditional resources), AI-only (ChatGPT-4o exclusively), or blended (ChatGPT-4o plus weekly faculty tutorials) groups. After a 3-week intervention, performance was assessed using a 150-item examination (pure-text and image-based multiple-choice questions). The study was approved by the institutional ethics committee, and written informed consent was obtained.

**Results:**

The blended group achieved the highest scores (mean 128.40 ± 18.25) vs. AI-only (119.87 ± 19.11) and control (110.60 ± 20.45; *P* = 0.02), with superior pure-text performance (*P* = 0.03) and significant advantages in obstetrics/gynaecology (*P* = 0.04) and superficial organ ultrasonography (*P* = 0.047). Examination time was shortest in the blended group (*P* = 0.03). ChatGPT-4o alone was 85% accurate on text but only 47% on image-based questions.

**Conclusions:**

A faculty-guided AI-integrated strategy was associated with improved short-term post-intervention performance compared with AI-only or traditional learning; however, effects reflect the combined intervention and AI support for static ultrasound image interpretation remains limited.

## Introduction

Ultrasonography is a cornerstone of modern diagnostic medicine, yet training residents remains challenging due to its operator-dependent nature, the need for extensive hands-on practice, and difficulties in mastering image interpretation of subtle grayscale nuances and anatomical relationships (1–3). These factors contribute to prolonged learning curves and variability in competency, particularly in resource-constrained settings.

Large language models (LLMs), such as ChatGPT, have emerged as potentially transformative tools in medical education by enabling rapid knowledge retrieval and explanation (4–6). However, their outputs may be variably accurate and can include omissions or hallucinations; therefore, educational use should be embedded within structured supervision and critical appraisal training, especially for image-based ultrasonography tasks. In radiology and related fields, multimodal versions like ChatGPT-4o demonstrate strong performance on text-based questions but marked limitations in interpreting static medical images, including ultrasonography (7–9). Hybrid approaches combining AI with human oversight have shown promise in enhancing learning outcomes across clinical disciplines (10).

Despite these advances, few randomized trials have evaluated AI-integrated ultrasonography training, particularly models blending LLM assistance with faculty guidance to address inaccuracies in image analysis and promote higher-order reasoning (11).

Accordingly, our objective was to compare traditional learning, ChatGPT-4o-only, and a pre-specified faculty-guided AI strategy (ChatGPT-4o plus debriefing) in first-year ultrasonography residents. We hypothesized that ChatGPT-4o-only would mainly enhance text-based knowledge, and that faculty-guided debriefing would yield incremental gains over AI-only, especially for image-related content.

## Methods

### Study design

This study is a prospective, single-center, randomized controlled trial with three groups: a control group, an AI-assisted learning group (AI group), and a blended learning group (Blended group). The control group used traditional resources, including textbooks and approved medical websites. The AI group used only ChatGPT-4o for knowledge retrieval and image analysis, while the Blended group used ChatGPT-4o and attended weekly 1-hour tutorial sessions led by an attending physician to evaluate and correct AI responses. Each group spent 1 h per day learning; adherence was monitored via weekly feedback forms, and non-adherence was defined *a priori* as missing >2 scheduled learning activities. In the blended arm, the weekly 1-hour faculty-led debriefing was conducted as part of the scheduled 1 h/day study time (i.e., it replaced one daily self-study session and did not add extra time).

### Participants

Participants were recruited from first-year residents in the Department of Ultrasonography at the Second Affiliated Hospital of Fujian Medical University during September–October 2025. The study coordinator invited all eligible first-year residents via departmental meetings and internal communications. A total of 45 residents provided written informed consent, completed a brief pre-enrolment screening questionnaire, and were enrolled. Eligibility was confirmed based on residency roster verification and the screening questionnaire (including prior LLM use frequency and anticipated schedule conflicts).

#### Inclusion criteria

(1) first-year ultrasonography residents undergoing standardized training in our department during the recruitment period; (2) provided written informed consent; (3) available to complete baseline assessment, the 3-week review period, and the post-intervention examination.

#### Exclusion criteria(pre-enrolment)

(1) regular use of LLMs (≥weekly) for ultrasonography learning; (2) anticipated schedule conflicts (e.g., planned leave/rotations) likely to preclude participation during the 3-week intervention.

#### Adherence (protocol definition)

Non-adherence was defined *a priori* as missing >2 scheduled learning activities during the intervention; participants meeting this criterion would be considered protocol deviations.

Eligibility (including prior LLM use) was screened using a brief pre-enrolment questionnaire administered by the study coordinator.

### Preparation

A set of ultrasonography-related questions was curated from the standardized resident training question bank of the Chinese Medical Association. Two attending ultrasonographers screened the questions. The scope of this examination covers three sub-specialties: Abdominal, Obstetrics/Gynecology, and Superficial Organ Ultrasonography. A total of 150 questions were selected, comprising 120 pure-text multiple-choice questions (MCQs) and 30 image-based MCQs, each question is worth one point, and the total score is 150 points. Chinese was used as the text input language for ChatGPT in this investigation. These questions were sequentially entered into ChatGPT-4o (OpenAI) in a new session for each question without additional context or prompt engineering, and its responses were recorded for accuracy analysis.

### Intervention

#### Initial study

All participants attended a series of three standardized lectures covering core modules of Abdominal, Obstetrics/Gynecology, and Superficial Organ Ultrasonography. Upon completion of the lectures, they underwent a baseline ultrasonography test. The lectures were standardized across participants using the same teaching materials and instructors. Both baseline and post-intervention examinations were administered via the same online testing system using the curated question bank.

#### Follow-Up study

A 3-week review period followed the initial lectures. Residents in the AI group were required to use ChatGPT-4o to search for knowledge points and analyze static ultrasonography images to aid their learning. The use of other web-based search engines or forums was prohibited. Residents in the Blended group used ChatGPT-4o under the same conditions but also participated in weekly, one-hour faculty-led debriefing sessions focusing on critical appraisal of AI-generated content and consolidation of key concepts, using a pre-specified checklist and the same faculty team across weeks. Residents in the control group were required to use traditional resources, including textbooks, clinical guidelines, and permitted search engines, to support their study. They were prohibited from using any OpenAI-related software or applications.

After the 3-week review period, all participants completed a post-intervention ultrasonography examination. To minimize contamination, participants were instructed not to share learning materials or AI-generated outputs across groups during the trial, and this was reinforced in the weekly feedback forms. Across the 3-week review period (21 days), the planned study time was 21 h in each arm (1 h/day). In the control arm, this comprised 21 h of self-study using traditional resources. In the AI-only arm, this comprised 21 h of AI-assisted self-study using ChatGPT-4o. In the blended arm, this comprised 18 h of AI-assisted self-study plus 3 h of faculty guidance (1 h/week × 3), all within the 21-hour schedule, replacing one daily self-study session each week rather than adding extra time. No additional hands-on practice sessions were mandated as part of the intervention protocol.

### Outcomes

In the preparation phase, we evaluated the accuracy of ChatGPT-4o in answering the curated ultrasonography MCQs as a preliminary analysis.

For the randomized trial, the primary outcome was the participants' performance on the post-intervention examination. The main indicators for assessment are the examination scores and the time taken to complete the examination (duration). The examination was administered without a strict time limit; however, the time each participant spent from starting until submitting the examination was automatically recorded by the online testing system. Examination duration was treated as a secondary efficiency proxy (time to finalize answers in a standardized test environment), rather than a direct measure of competence.

### Blinding

To eradicate subjective bias in the grading process, the collectors and graders who assessed the post-intervention examinations were unaware of the group allocation of the participants.

### Randomization

After the baseline test, an independent statistician generated a 1:1:1 random allocation sequence (computer-generated). Allocation was concealed using sequentially numbered, opaque, sealed envelopes prepared by a staff member not involved in enrolment or assessment. After enrolment and baseline assessment, the study coordinator opened the next envelope in sequence to assign participants. The envelope set was stored securely and opened only after participant enrolment and completion of baseline testing.

### Statistics

Because the eligible first-year resident cohort was fixed, we planned to enroll all available participants during the recruitment period (*n* = 45; 15 per arm). With *n* = 45 and three groups, the study has 80% power to detect a large omnibus effect (Cohen's f = 0.48, equivalent to *η*^2^ ≈ 0.19) at *α* = 0.05. Accordingly, analyses of secondary outcomes should be interpreted as exploratory and may be underpowered for small-to-moderate effects. For normally distributed data with homogeneous variance (Levene's test), we used one-way ANOVA and reported F statistics (df1, df2), two-sided *P* values, and effect sizes ($\eta_{p}^{2}$). Pairwise comparisons were performed using Tukey's HSD (family-wise error controlled). If variance heterogeneity was present, we used Welch's ANOVA with Games–Howell *post hoc*. For non-normal data, we used Kruskal–Wallis and reported H (df) and *ε*^2^.

### Ethics statement

This study was approved by the Ethics Committee of Second Affiliated Hospital of Fujian Medical University (No. ([2025]) 624). In accordance with the Declaration of Helsinki, written informed consent was obtained from all participants before the study commenced. To ensure confidentiality, access to the original experimental data requires a reasonable request sent to the corresponding author's email address.

## Results

### Overview

**Figure 1:**
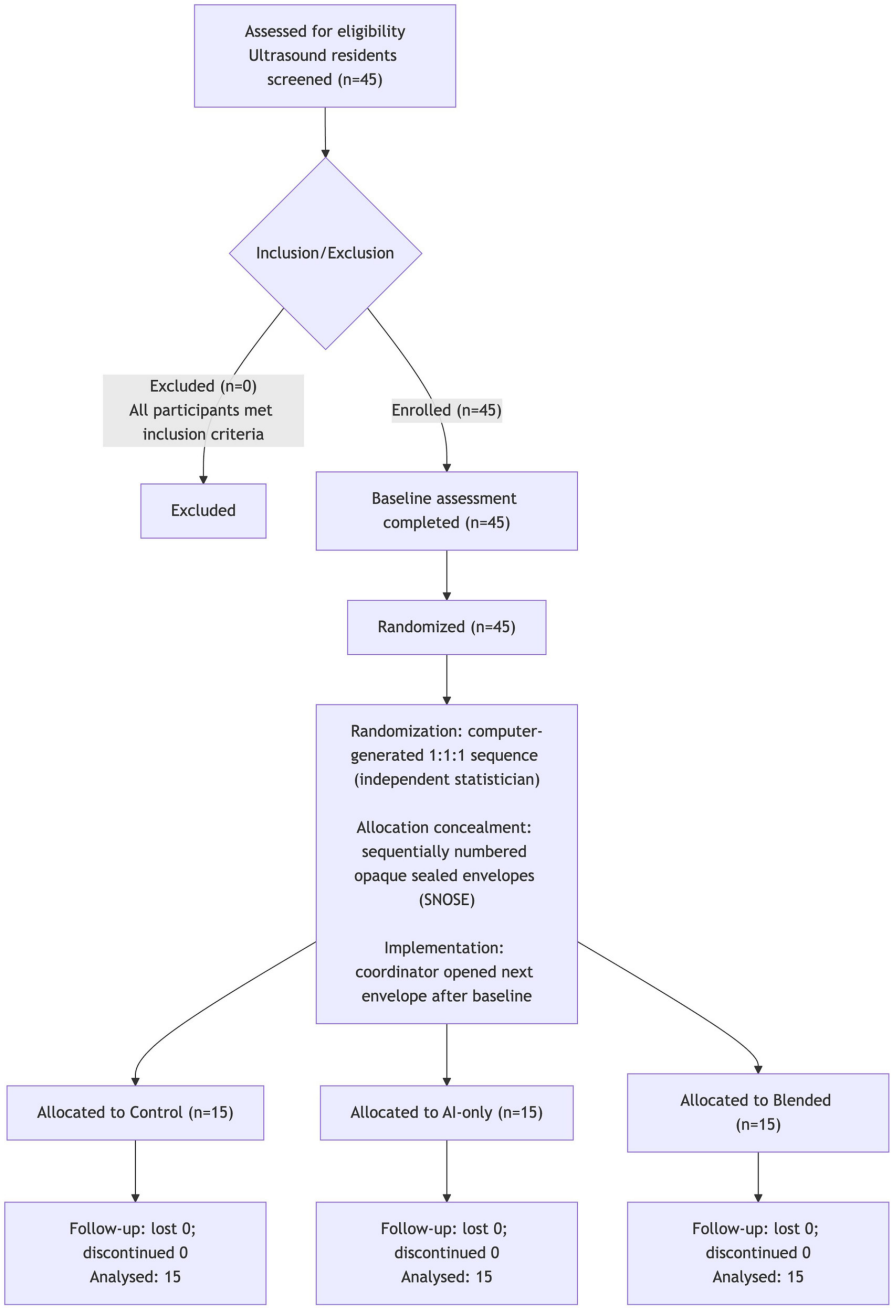
CONSORT flow diagram.

We began recruiting first-year residents from the Department of Ultrasonography on September 1, 2025, and finished the recruitment process on October 31, 2025. All 45 recruited participants were randomized into the three groups. During the 3-week intervention period, no participants withdrew from the study. All randomized participants (15 per group) completed the intervention and post-test and were included in the primary analysis(see Figure 1). The baseline characteristics of the participants are presented in Table 1. There were no statistically significant differences in age, gender, or baseline test scores among the three groups [gender: *χ*^2^(2) = 0.73, *P* = 0.695, Cramer's V = 0.127; age: Kruskal–Wallis H(2) = 3.14, *P* = 0.208, *ε*^2^=0.027; baseline score: one-way ANOVA F(2,42) = 0.40, *P* = 0.671, $\eta_{p}^{2}=0.019$], indicating that the groups were well-balanced at the outset.

**Table 1:** Baseline characteristics of the participants.

Characteristics	Control group (*n* = 15)	AI group (*n* = 15)	Blended group (*n* = 15)	*P* value
Age (years), mean (SD)	27.2 (1.3)	27.0 (1.1)	26.9 (1.4)	0.208
Male sex, *n* (%)	7 (46.7)	6 (40.0)	8 (53.3)	0.695
Baseline test score, mean (SD)	68.41 (5.23)	67.85 (6.10)	69.02 (5.67)	0.671

### The accuracy of ChatGPT-4o responses to ultrasonography-related MCQs

A total of 150 ultrasonography-related MCQs were input into ChatGPT-4o for evaluation. The overall accuracy rate was 73.3% (110/150). Specifically, for the 120 pure-text MCQs, the accuracy was 85.0% (102/120). In contrast, for the 30 image-based questions featuring static ultrasonography images, the accuracy was significantly lower at 46.7% (14/30). We observed that ChatGPT-4o provided explanatory reasoning for its choices in both text and image-based queries.

### The performance of the three participant groups in the post-intervention ultrasonography examination

**Figure 2:**
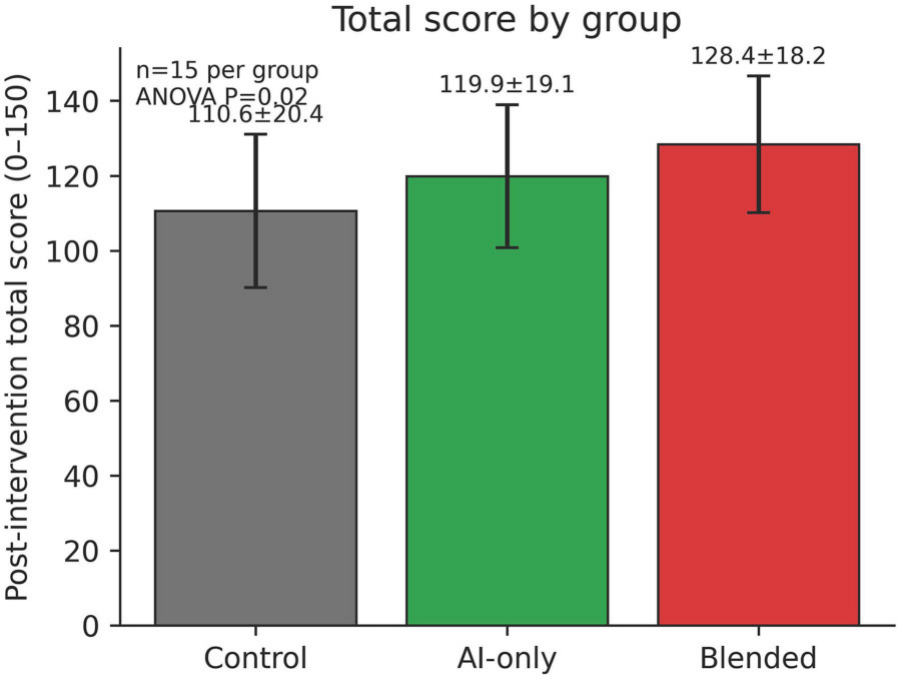
Post-intervention total scores across the three study groups.

**Figure 3:**
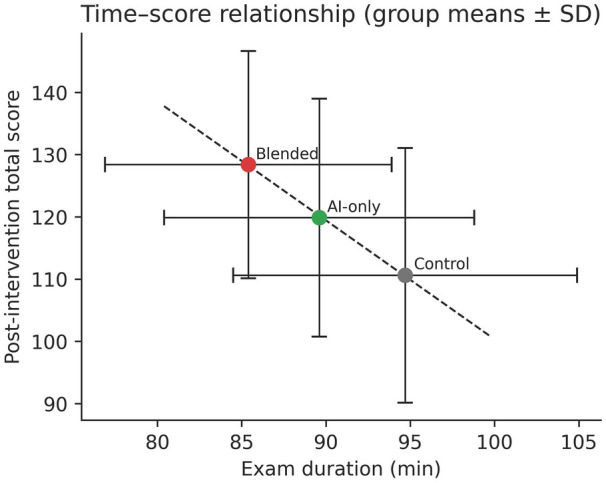
Relationship between exam duration and post-intervention total score by group.

In the post-intervention examination, we found that the number of correctly answered questions differed significantly among the three groups [Blended group: mean 128.40, SD 18.25; AI group: mean 119.87, SD 19.11; Control group: mean 110.60, SD 20.45; Welch's ANOVA F(2.00, 26.29) = 12.13, *P* = 0.02, $\eta_{p}^{2}=0.367$; *post hoc*: Games-Howell]. As shown in Figure 2, this difference was primarily driven by the performance on the pure-text MCQs [Blended group: mean 104.20, SD 14.80; AI group: mean 98.50, SD 15.60; Control group: mean 90.30, SD 16.90; one-way ANOVA F(2,42) = 6.74, *P* = 0.0029, $\eta_{p}^{2}=0.243$; *post hoc*: Tukey HSD], where both the Blended and AI groups outperformed the control group. The performance on the image-based MCQs also showed a similar trend, although the differences were not statistically significant [Blended group: mean 24.20, SD 5.10; AI group: mean 21.37, SD 5.80; Control group: mean 20.30, SD 6.20; one-way ANOVA F(2,42) = 3.16, *P* = 0.0528, $\eta_{p}^{2}=0.131$]. In the comparison of the duration of the exam, the Blended group (mean 85.4 min, SD 8.5) was shorter than the AI group (mean 89.6 min, SD 9.2), which in turn was shorter than the Control group [mean 94.7 min, SD 10.2; one-way ANOVA F(2,42) = 6.12, *P* = 0.00466, $\eta_{p}^{2}=0.226$; *post hoc*: Tukey HSD] (Figure 3). Figure 3 illustrates the relationship between exam duration and post-intervention total score across groups.

**Figure 4:**
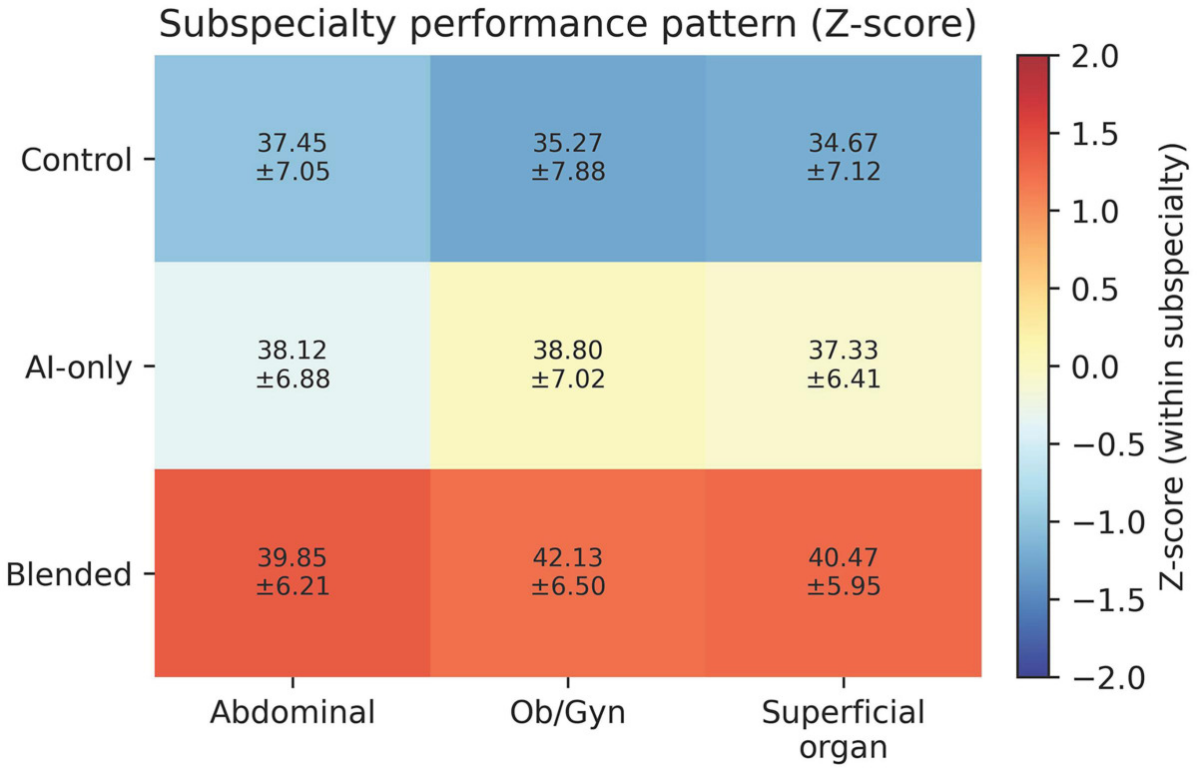
Heatmap of subspecialty performance across groups (Z-score normalized).

Subsequently, we analyzed the examination scores by ultrasonography subspecialties (Figure 4). We found that for both Obstetrics/Gynecology Ultrasonography [Blended group: mean 42.13, SD 6.50; AI group: mean 38.80, SD 7.02; Control group: mean 35.27, SD 7.88; one-way ANOVA F(2,42) = 4.08, *P* = 0.0412, $\eta_{p}^{2}=0.126$] and Superficial Organ Ultrasonography [Blended group: mean 40.47, SD 5.95; AI group: mean 37.33, SD 6.41; Control group: mean 34.67, SD 7.12; one-way ANOVA F(2,42) = 7.01, *P* = 0.00235, $\eta_{p}^{2}=0.250$; *post hoc*: Tukey HSD], the Blended group scored significantly higher than the AI group, which in turn scored higher than the Control group. For Abdominal Ultrasonography, the Blended group showed higher mean scores than the other two groups, but the difference was not statistically significant (*P* = 0.41) (Figure 4, Table 2). Details of the statistical tests and effect sizes are provided in Table 3.

**Table 2:** Post-intervention examination scores across different subspecialties and assessment types.

Domain/subspecialty	Control group (*n* = 15)	AI group (*n* = 15)	Blended group (*n* = 15)	*P* value
Pure-text MCQs	90.30 (16.90)	98.50 (15.60)	104.20 (14.80)	0.0029
Image-based MCQs	20.30 (6.20)	21.37 (5.80)	24.20 (5.10)	0.0528
Obstetrics/Gynecology	35.27 (7.88)	38.80 (7.02)	42.13 (6.50)	0.0412
Superficial Organ	34.67 (7.12)	37.33 (6.41)	40.47 (5.95)	0.0023
Abdominal	37.45 (7.05)	38.12 (6.88)	39.85 (6.21)	0.25

**Table 3:** Statistical transparency summary (tests, statistics, effect sizes, and *post hoc* comparisons).

Outcome	Primary test	Test statistic (df)	*P* value	Effect size (95% CI)	Post hoc (adjusted *P* < 0.05)
Gender (Male/Female)	Chi-square	*χ*^2^ (2) = 0.73	0.695	Cramer's V = 0.127	—
Age (years)	Kruskal–Wallis	H(2) = 3.14	0.208	*ε*^2^ = 0.027	—
Baseline test score	One-way ANOVA	F(2,42) = 0.40	0.671	$\eta_{p}^{2}=0.019$	—
Post-test total score	Welch ANOVA	F(2.00,26.29) = 12.13	0.0,00,186	$\eta_{p}^{2}=0.480$ (95% CI 0.176–0.662)	Games–Howell: Control vs Blended; AI-only vs Blended
Post-test pure-text MCQs	One-way ANOVA	F(2,42) = 6.74	0.0029	$\eta_{p}^{2}=0.243$ (95% CI 0.039–0.432)	Tukey HSD: Blended vs Control
Post-test image-based MCQs	One-way ANOVA	F(2,42) = 3.16	0.0528	$\eta_{p}^{2}=0.131$ (95% CI 0.000–0.314)	—
Exam duration (min)	One-way ANOVA	F(2,42) = 6.12	0.00466	$\eta_{p}^{2}=0.226$ (95% CI 0.029–0.416)	Tukey HSD: Blended vs AI-only; Blended vs Control
Obstetrics/Gynecology subscore	One-way ANOVA	F(2,42) = 4.08	0.0412	$\eta_{p}^{2}=0.126$	Tukey HSD: AI-only vs Control; Blended vs Control
Superficial Organ subscore	One-way ANOVA	F(2,42) = 7.01	0.00235	$\eta_{p}^{2}=0.250$	Tukey HSD: AI-only vs Control; Blended vs Control
Abdominal subscore	Kruskal–Wallis	H(2) = 2.77	0.25	ε^2^ = 0.018	—

Post hoc comparisons were conducted only when the omnibus test was significant (*P* < 0.05).

Effect sizes: $\eta_{p}^{2}$ for ANOVA/Welch ANOVA; ε^2^ for Kruskal–Wallis; Cramer's V for χ^2^. 95% CIs are reported for primary post-intervention outcomes.

For *post hoc* results, only comparisons with adjusted *P* < 0.05 are listed to keep the table concise.

## Discussion

In this randomized trial, we evaluated learner outcomes under three study strategies and observed higher post-intervention scores in the AI-only and blended strategies than in the control group. In a preliminary item-level evaluation using our curated MCQ bank, ChatGPT-4o answered text items more often correctly than image-based items, suggesting that learners may benefit more from AI support for text-based consolidation than for static image interpretation. In the post-intervention assessment, the blended strategy (ChatGPT-4o plus faculty debriefing) was associated with the highest scores; however, this effect reflects the combined intervention rather than ChatGPT-4o alone.

Our findings elucidate several key insights regarding the integration of LLMs into specialized medical education. Consistent with prior studies, ChatGPT-assisted learning can support short-term knowledge acquisition when used with appropriate oversight (4, 5, 12–14). The blended-group advantage likely reflects the combined intervention (ChatGPT-4o plus faculty debriefing), and individual contributions cannot be separated. While ChatGPT-4o serves as a powerful tool for rapid information retrieval and providing instant, detailed explanations—effectively breaking the traditional one-way transmission of knowledge—it is not infallible (15). In our study, faculty-led debriefing focused on critical appraisal and correction of AI outputs, which may reduce the risk of internalizing AI errors. This hybrid approach effectively mitigates the risk of students internalizing AI-generated errors, a significant concern when using LLMs autonomously.

The lower performance on image-based questions suggests that AI support for static ultrasound image interpretation remains limited and should be complemented by faculty supervision (7, 8, 16–18). This may relate to the difficulty of extracting subtle grayscale patterns and anatomical relationships from static ultrasound images without clinical context. This inherent limitation highlights that current LLMs are better suited as aids for theoretical consolidation than for primary image diagnosis training. Educationally, this suggests that AI outputs should not be used as a primary signal for ultrasound image diagnosis training. Instead, AI may be best positioned to support conceptual frameworks and differential diagnosis checklists, while image reasoning should remain anchored in supervised faculty feedback.

Interestingly, the Blended learning model demonstrated its most significant advantages in the subspecialties of Obstetrics/Gynecology and Superficial Organ Ultrasonography. We postulate that the knowledge and diagnostic criteria in these areas, such as the assessment of fetal biometry, thyroid nodules, or breast lesions, are often highly standardized and richly detailed in textual literature. This structured nature makes the knowledge more readily accessible and accurately collatable by ChatGPT-4o. Consequently, residents can use the AI to efficiently build a robust theoretical framework, which is then refined and applied to image interpretation under the guidance of an instructor, maximizing learning efficiency in these particular domains (19). Notably, the significant reduction in examination completion time observed in the Blended group, as compared to the AI-only and Control groups, Shorter completion time may reflect faster information retrieval and decision finalization under testing conditions, but it should be interpreted cautiously as it is not a direct educational endpoint.

Consistent with prior studies, ChatGPT-assisted learning can support short-term knowledge acquisition when used with appropriate oversight (4, 5, 12–14). Within ultrasound education, the need for structured supervision and careful integration of digital tools—including AI—has been emphasized, given the image-dependent nature of training and ongoing debates about best instructional formats (20). Blended approaches that combine digital preparation with structured, faculty-guided feedback have also been reported as feasible and beneficial in ultrasound-related skills training (21). In our study, faculty-led debriefing focused on critical appraisal and correction of AI outputs, which may reduce the risk of internalizing AI errors (15).

Overall, our results support the feasibility of a faculty-guided AI-integrated study strategy for short-term examination performance in first-year ultrasonography residents. Emerging ultrasound curricula that incorporate AI-supported teaching and blended e-learning have been evaluated in randomized or multicenter pilot formats, providing a useful point of comparison for integrating AI tools within structured educational design (22). These findings suggest that AI tools may be most useful when embedded within an instructional framework that emphasizes verification and feedback, offering a practical approach to leveraging AI's efficiency while supporting the development of clinical reasoning. Accordingly, implementation may require adaptation of the educator's role, along with targeted faculty training and standardized prompts to guide critical appraisal of AI outputs. However, given the single-center design, small sample size, and short follow-up, our findings should not be generalized to clinical scanning competence, diagnostic accuracy, or long-term retention. Larger multicenter studies with longer follow-up and factorial designs are warranted to isolate the marginal effects of AI access and faculty feedback. Because faculty guidance constitutes an additional instructional modality, the blended effect should not be attributed to AI alone even with equivalent scheduled study time.

This study has several limitations. First, although the planned study time was equivalent across arms (1 h/day for 3 weeks), the blended arm included faculty-led debriefing, and thus any incremental benefit should be interpreted as the effect of a combined strategy rather than AI alone. Second, contamination between groups cannot be completely excluded despite instructions and weekly reinforcement not to share AI outputs or learning materials. Despite these measures, informal peer-to-peer discussion and inadvertent sharing of study approaches could have occurred and may have attenuated between-group differences. Third, this was a single-center study with a small sample of first-year residents and short follow-up; the findings may not generalize to other institutions, training levels, or long-term retention. Finally, outcomes were based on an MCQ-style assessment (including static-image items), which measures short-term examination performance rather than hands-on scanning competence or real-world diagnostic accuracy.

## Conclusions

AI-assisted learning strategies were associated with improved short-term post-intervention examination performance compared with traditional resources in first-year ultrasonography residents. The highest scores were observed in the faculty-guided AI-integrated (blended) strategy; however, any blended-arm advantage should be interpreted as the effect of the combined strategy (AI plus faculty debriefing) rather than ChatGPT-4o alone, even though total scheduled study time was equivalent across groups. AI support for static ultrasound image interpretation remained limited, suggesting that AI outputs should be used as a supplementary aid with verification and faculty feedback for imaging-related learning. Larger multicenter studies with longer follow-up are needed to assess durability, generalizability, and effects on hands-on scanning competence.

## Data Availability

The original contributions presented in the study are included in the article/Supplementary Material, further inquiries can be directed to the corresponding author.
